# Case report: Thyroid storm in a three-year-old girl presenting with febrile status epilepticus and hypoglycemia

**DOI:** 10.3389/fped.2023.1213040

**Published:** 2023-06-16

**Authors:** Yusuke Aoki, Ryo Hanaki, Hidemi Toyoda, Koichi Emori, Masazumi Miyahara, Masahiro Hirayama

**Affiliations:** ^1^ Department of Pediatrics, Mie University Graduate School of Medicine, Tsu, Japan; ^2^ Department of Pediatrics, Okanami General Hospital, Iga, Japan

**Keywords:** Graves' disease, hyperthyroid, thyroid storm, febrile status epilepticus, febrile convulsion, hypoglycemia, beta 1 blocker

## Abstract

Thyroid storm, though extremely rare in toddlers, requires prompt diagnosis and treatment because it can be fatal if left untreated. However, thyroid storm is not often considered in the differential diagnosis of a febrile convulsion due to its rarity in children. Herein, we report the case of a 3-year-old girl with thyroid storm who presented with febrile status epilepticus. Although the seizure was stopped by diazepam administration, her tachycardia and widened pulse pressure persisted, and severe hypoglycemia was observed. Based on the findings of thyromegaly, a history of excessive sweating and hyperactivity, and a family history of Graves' disease, she was eventually diagnosed with a thyroid storm. The patient was successfully treated with thiamazole, landiolol, hydrocortisone, and potassium iodide. Propranolol, a non-selective *β*-blocker, has been used to manage tachycardia during thyroid storm. However, a cardio-selective *β*1-blockers, landiolol hydrochloride, was used in our case to avoid worsening hypoglycemia. Febrile status epilepticus is one of the most common medical emergencies in childhood; it is necessary to rule out treatable underlying critical diseases such as septic meningitis and encephalitis. Thyroid storm should be considered in children presenting with prolonged febrile convulsion accompanied by findings that are not usually observed with febrile convulsions.

## Introduction

A febrile convulsion is defined as a seizure that occurs in children aged 6 through 60 months in the presence of a fever (temperature of 38 °C or higher) (1). Febrile convulsions occur in 2% to 5% of all children and are the most common convulsive event in children <5 years (1). However, it is imperative to rule out any underlying critical condition that may require further intervention and treatment, such as central nervous system infections (meningitis, encephalitis, and brain abscess), metabolic disturbance, and trauma.

Thyroid storm is a severe manifestation of Graves' disease that can lead to multi-organ failure with a high mortality rate if not promptly detected (2, 3). Common clinical features of thyroid storm include high fever, dehydration, marked tachycardia or tachyarrhythmias, heart failure, respiratory distress, abdominal pain, delirium, and possibly seizures (3–5). Although pediatricians and emergency physicians consider central nervous system infections as possible underlying causes in cases of febrile convulsions, thyroid storm is not typically considered in the differential diagnosis.

Management of patients in thyroid storm should be conducted in a high or intensive care setting, with a multimodality treatment approach, which includes antithyroid drugs, inorganic iodide, corticosteroid, and *β*-adrenergic blockade. Antithyroid drugs and iodine block thyroid hormone synthesis and secretion, while glucocorticoids inhibit the peripheral conversion of the inactive prohormone T4 to the metabolically active T3 (6). *β*-blockers are essential in controlling the peripheral actions of thyroid hormones and a rapid heart rate in patients with thyroid storm. Despite the historic use of propranolol, a non-selective *β*-blocker, in treating thyroid storm, great caution should be exercised when hypoglycemia is a concern because propranolol is thought to impair normal glucose homeostasis through inhibition of *β*-adrenergic mediated glycogenolysis and gluconeogenesis (7).

Herein, we report the case of thyroid storm in possibly the youngest patient. The patient initially presented with a febrile status epilepticus and hypoglycemia and was successfully treated with thiamazole; landiolol hydrochloride, an ultra-short-acting cardio-selective *β*1-blocker; hydrocortisone; and potassium iodide solution.

## Case presentation

**Figure 1:**
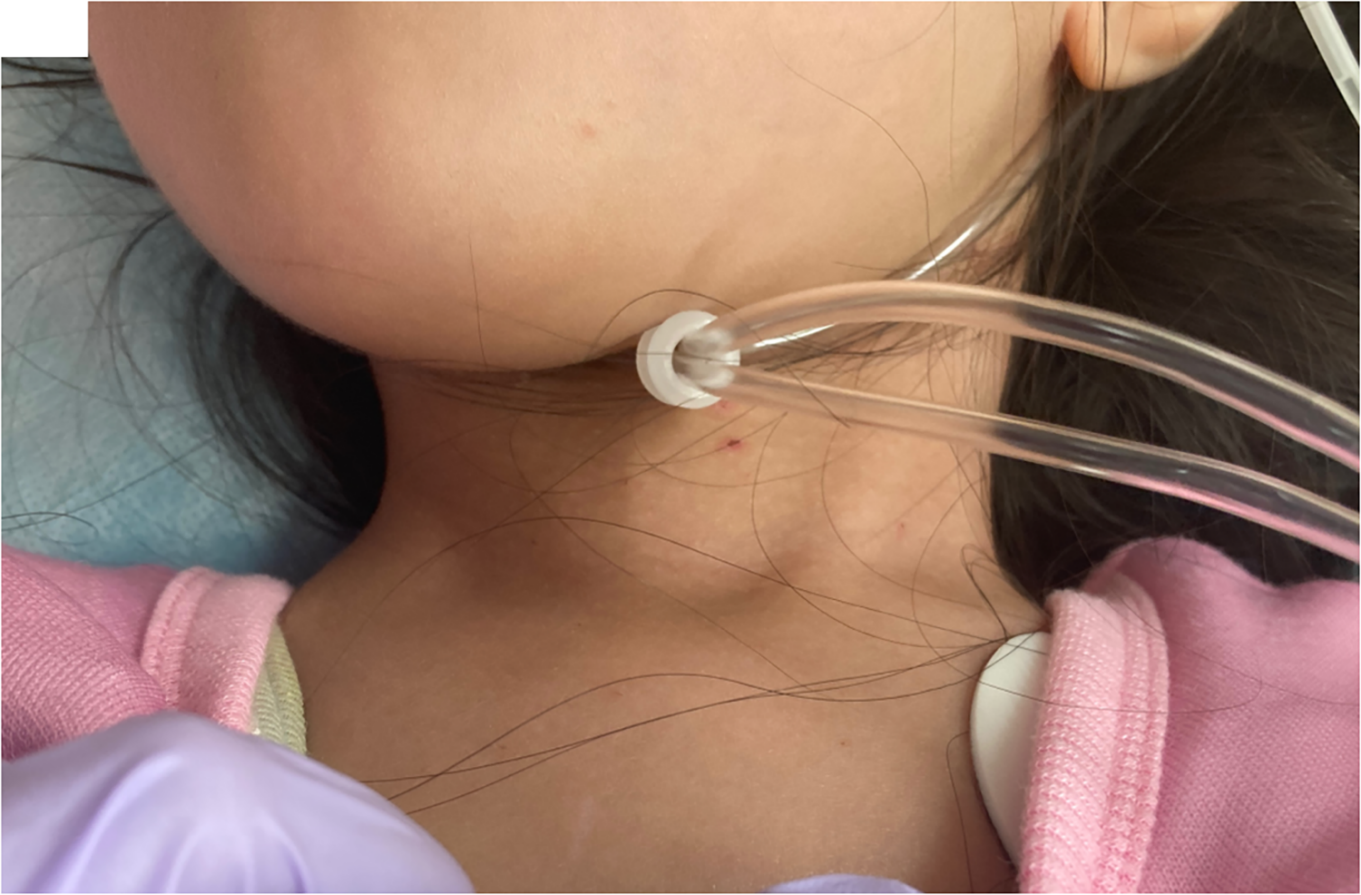
Photograph of the patient on admission showing an enlarged and homogeneous thyroid.

A 3-year and 7 months old girl was brought to the emergency department because of unconsciousness, full-body stiffness, and clonic movements lasting for 1 h. Her past medical history was unremarkable. On arrival, vital signs revealed fever of 40.3 °C, tachycardia with a heart rate of 208 bpm, and elevated systolic blood pressure with widened pulse pressure (123/46 mmHg). The patient weighed 14 kg (50th percentile) with a height of 99.7 cm (84th percentile). She was experiencing status epilepticus with generalized tonic–clonic seizures lasting for 1 h, which were treated with diazepam administration in the emergency department. Postictal clinical examination revealed no paresis. Laboratory investigation revealed an elevated anion gap metabolic acidosis (capillary blood gas pH, 7.21; pCO_2_, 37.2 mmHg; bicarbonate, 14.5 mmol/L; anion gap, 22.7 mmol/L; lactate, 1.4 mmol/L), severe hypoglycemia (13 mg/dl), an extremely high white blood cell count (39.9 × 10^9^/L) with a neutrophil predominance (87%). No meningeal irritation symptoms were noted; however, a lumbar puncture was performed. Cerebrospinal fluid (CSF) analysis revealed no abnormality. Rapid antigen tests for severe acute respiratory syndrome coronavirus 2 (SARS-CoV-2) and influenza were negative. The patient's hypoglycemia was corrected with an intravenous injection of dextrose solution; however, she remained unwell and was admitted for further management of status epilepticus, continued tachycardia, metabolic acidosis, and hypoglycemia. On detailed history taking, the patient's mother reported sweating and hyperactivity for 2 years before the admission and a significant family history of the patient's father being treated for Graves' disease for 5 years. Repeat examination on admission revealed thyromegaly (Figure 1) and a holosystolic murmur best heard at the apex, without hepatomegaly or adventitious lung sounds. Serum thyroid function tests revealed suppressed thyroid-stimulating hormone (TSH) levels of <0.01 mIU/L (reference range 0.35–4.94 mIU/L), elevated free thyroxine (free T4) levels of >5.0 ng/dl (reference range 0.70–1.48 ng/dl), and free triiodothyronine (free T3) levels of > 20.0 pg/ml (reference range 1.68–3.67 pg/ml). Anti–TSH-receptor antibodies and anti-thyroid peroxidase antibodies were markedly positive at 44.1 IU/L (reference range 0–2 IU/L) and 190 IU/ml (reference range 0–16 IU/L), respectively. Additional examinations revealed brain natriuretic peptide levels of 89.2 pg/ml (reference range 0–18.4 pg/ml). An electrocardiogram revealed sinus tachycardia, while chest radiograph showed clear lung fields with mild cardiomegaly. A transthoracic echocardiogram showed moderate mitral regurgitation. Blood, CSF, urine, and stool cultures were all negative. Based on the clinical and laboratory findings, the patient was diagnosed with Graves' disease, and based on the criteria of hyperpyrexia, tachycardia, and seizures, she was experiencing a thyroid storm (8, 9).

**Figure 2:**
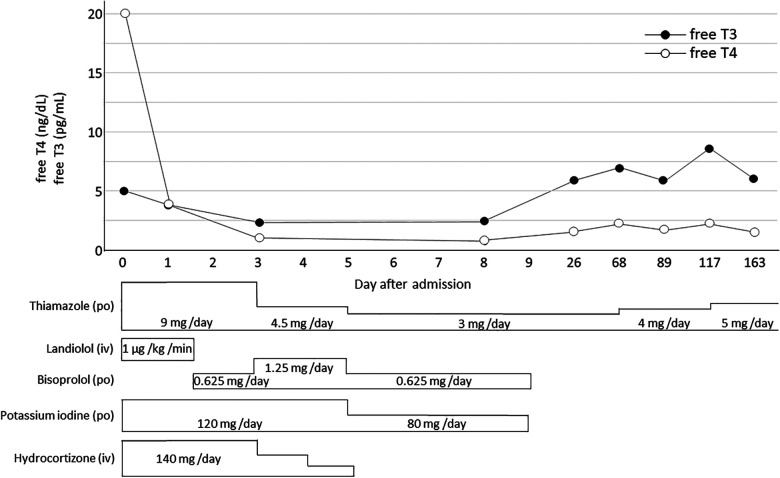
The patient's thyroid hormone levels (free T4 and free T3) trend over time iv, intravenous; po, per os.

The patient was initially started on thiamazole (9 mg/day), landiolol hydrochloride (1 μg/kg/min), and hydrocortisone (140 mg/day). Potassium iodide solution (120 mg/day) was subsequently added (Figure 2). The patient's vital signs and thyroid function tests slowly improved (Figure 2). Intravenous landiolol hydrochloride was replaced by oral bisoprolol fumarate on day 2 of admission, and on day 7, the potassium iodide solution was discontinued. Hydrocortisone was weaned over the course of 5 days, while bisoprolol fumarate was weaned after 10 days. The patient was discharged from the hospital on day 10 with home medication of oral thiamazole, 1.5 mg, to be taken every 12 h. Two months after presentation, her thiamazole dose was increased for hyperthyroidism control (free T4 of 2.20 ng/dl) and then titrated as necessary on an outpatient basis (Figure 2). At 1-year follow-up, our patient remained clinically well and on thiamazole monotherapy with no recurrences of seizure.

## Discussion

Thyroid storm is a rare but severe manifestation of Graves' disease and is considered an endocrine emergency, as it can be fatal if not detected and treated promptly. The diagnosis of thyroid storm is difficult, especially in undiagnosed patients with Graves' disease, because it may mimic sepsis, heat stroke, gastrointestinal infection, or febrile convulsion. Although the incidence of thyroid storm in pediatric Graves' disease is not well established, it is likely rare, with only case reports and case series reported in the literature, mostly in school-aged to teen-aged children (median age 11.25 years) (4). Studies with populations including children and adults showed that only 0.2% to 4% of patients with thyroid storm presented with seizures (10, 11). In our review of the pediatric literature, we identified 15 reported cases of seizures in children with endogenously caused thyroid storm (3, 12–21), suggesting that seizures may occur more often among children experiencing thyroid storm than among adults. Nonetheless, to the best of our knowledge, no study has reported febrile status epilepticus due to thyroid storm.

Hypoglycemia co-occurring with thyroid storm is quite rare, with only seven cases reported in adults and none in children (22–28). In cases of thyroid storm, congestive heart failure and severe liver dysfunction have been reported as causes of hypoglycemia (29). Although we could not obtain sufficient evidence to pinpoint the exact cause of the hypoglycemia in our case, it could have been caused by relative adrenal failure, known to accompany thyroid storm.

Thyroid storm is clinically diagnosed after confirming the presence of hyperthyroidism from thyroid function tests. Although not specific to pediatrics, patients with a Burch–Wartofsky Point Scale of ≥45 or Japanese Thyroid Association categories of thyroid storm 1 (TS1) or thyroid storm 2 (TS2), with evidence of systemic decompensation, require urgent therapy to block new thyroid hormone synthesis and achieve symptomatic relief (8). The American Thyroid Association guidelines prefer the anti-thyroid medication propylthiouracil over thiamazole, considering that propylthiouracil both blocks new thyroid hormone synthesis and prevents the conversion of thyroxine to the more biologically active T3 (8). However, we utilized thiamazole to treat hyperthyroidism in our case because propylthiouracil increases the risk of hepatic failure; furthermore, thiamazole is not inferior to propylthiouracil (6, 9). Following guidelines, a saturated potassium iodide solution was added to prevent new thyroid hormone synthesis, while hydrocortisone prevented T4-to-T3 conversion and protected against relative adrenal insufficiency. *β*-blockers are recommended for the management of tachycardia during thyroid storm (6, 8). Propranolol has been used to manage thyroid storm, but worsening heart failure and cardiac arrest have been previously reported when using propranolol in such cases (30). Furthermore, the use of non-selective *β*-blocker propranolol was avoided due to the risk of worsening hypoglycemic control because our patient was hypoglycemic. Thus, we intravenously administered landiolol hydrochloride, a novel form of ultra-short-acting cardio-selective *β*1-blockers, and subsequently switched to oral bisoprolol, a *β*1-selective blocker. In our case, hypoglycemia was not observed during landiolol hydrochloride and bisoprolol treatment.

In conclusion, we report one of the youngest patients with thyroid storm who initially presented with febrile status epilepticus and hypoglycemia and was successfully treated with thiamazole, landiolol hydrochloride, hydrocortisone, and potassium iodide solution. Pediatricians should consider thyroid storm in children who present with prolonged febrile seizure if there are findings that are not usually seen with febrile convulsions, such as persisting tachycardia, widened pulse pressure, and hypoglycemia because this condition requires emergency treatment.

## Data Availability

The raw data supporting the conclusions of this article will be made available by the authors, without undue reservation.
